# Synthesis, Density Functional Theory (DFT), Urease Inhibition and Antimicrobial Activities of 5-Aryl Thiophenes Bearing Sulphonylacetamide Moieties

**DOI:** 10.3390/molecules201119661

**Published:** 2015-11-05

**Authors:** Mnaza Noreen, Nasir Rasool, Yasmeen Gull, Muhammad Zubair, Tariq Mahmood, Khurshid Ayub, Faiz-ul-Hassan Nasim, Asma Yaqoob, Muhammad Zia-Ul-Haq, Vincenzo de Feo

**Affiliations:** 1 Department of Chemistry, Government College University Faisalabad, Faisalabad 38000, Pakistan; mnazanoreen@yahoo.com (M.N.); yasmeenchem1@yahoo.com (Y.G.); zubairmkn@yahoo.com (M.Z.); 2 Department of Chemistry, COMSATS Institute of Information Technology, University Road, Tobe Camp, Abbottabad 22060, Pakistan; mahmood@ciit.net.pk (T.M.); khurshid@ciit.net.pk (K.A.); 3 Department of Chemistry, The Islamia University of Bahawalpur, Bahawalpur 63000, Pakistan; faiznasim@hotmail.com (F.-H.N.); asmayaqoobctn@gmail.com (A.Y.); 4 The Patent Office, Karachi 74200, Pakistan; ahirzia@gmail.com; 5 Department of Pharmaceutical and Biomedical Sciences, University of Salerno, Via Ponte don Melillo, Fisciano (Salerno) I-84084, Italy

**Keywords:** sulfacetamide, Suzuki cross coupling reactions, urease activity, antibacterial activity, DFT, frontier molecular orbitals (FMOs) analysis, MEP, hyperpolarizability and non-linear optical (NLO) properties

## Abstract

A variety of novel 5-aryl thiophenes **4a**–**g** containing sulphonylacetamide (sulfacetamide) groups were synthesized in appreciable yields via Pd[0] Suzuki cross coupling reactions. The structures of these newly synthesized compounds were determined using spectral data and elemental analysis. Density functional theory (DFT) studies were performed using the B3LYP/6-31G (d, p) basis set to gain insight into their structural properties. Frontier molecular orbital (FMOs) analysis of all compounds **4a**–**g** was computed at the same level of theory to get an idea about their kinetic stability. The molecular electrostatic potential (MEP) mapping over the entire stabilized geometries of the molecules indicated the reactive sites. First hyperpolarizability analysis (nonlinear optical response) were simulated at the B3LYP/6-31G (d, p) level of theory as well. The compounds were further evaluated for their promising antibacterial and anti-urease activities. In this case, the antibacterial activities were estimated by the agar well diffusion method, whereas the anti-urease activities of these compounds were determined using the indophenol method by quantifying the evolved ammonia produced. The results revealed that all the sulfacetamide derivatives displayed antibacterial activity against *Bacillus subtiles*, *Escherichia coli*, *Staphylococcus aureus*, *Shigella dysenteriae*, *Salmonella typhae*, *Pseudomonas aeruginosa* at various concentrations. Furthermore, the compound **4g** *N*-((5-(4-chlorophenyl)thiophen-2-yl)sulfonyl) acetamide showed excellent urease inhibition with percentage inhibition activity ~46.23 ± 0.11 at 15 µg/mL with IC_50_ 17.1 µg/mL. Moreover, some other compounds **4a**–**f** also exhibited very good inhibition against urease enzyme.

## 1. Introduction

*N*-acylsulfonamide (sulfacetamide) is well known basic common structural motif [1], which is present as a functional group in a wide range of therapeutics [2]. The formation of *N*-acylsulfonamides (sulfacetamides) is synthetically important for easy access to various structures [3]. Molecules containing acylsulfonamide (sulfacetamide) functional groups have been explored as HCV protease inhibitors and CXCR_2_ antagonists [4]. Moreover aryl sulfonamides are potential therapeutic agents in a number of pharmaceutical patents with a wide range of biological activities [5]. The *N*-acylsulfonamide (sulfacetamide) moiety has great importance in drug chemistry due to its various biological activities. Sulfonamide –SO_2_NH– bearing compounds are biologically active [6]. Therefore, we focused on the synthesis of 5-bromothiophene-2-sulfonamide followed by the *N*-acylation of 5-bromothiophene-2-sulfonamide followed by Suzuki cross coupling to obtain various 5-arylthiophene-2-sulfonylacetamide derivatives. The inspiration of this work was the very little attention paid tothe Pd[0] catalyzed Suzuki cross coupling reactions of *N*-acylsulfonamides (sulfacetamides). The major advantages of the Suzuki cross coupling reaction compared to other coupling reactions is the ready availability of different aryl boronic acids/esters [7]. Moreover, these reactions can be carried out under mild conditions compared to other organometallic reactions [1]. The newly synthesized compounds were isolated and characterized by ^1^H-NMR, ^13^C-NMR and mass spectrometry [8,9]. Moreover, these compounds were investigated for their structure activity relationship by using density functional theory (DFT), and evaluated for urease inhibition and antibacterial activities.

## 2. Results and Discussion

### 2.1. Chemistry

**Scheme 1:**
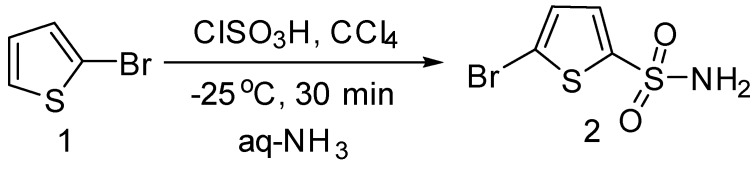
Synthesis of 5-bromothiophene-2-sulfonamide (**2**). *Reagents and conditions*: Bromothiophene (12 mmol), chlorosulfonic acid (40–60 mmol), solvent (CCl_4_, 6 mL).

Herein, we report the synthesis of a series of new 5-bromothiophene-2-sulphonylacetamidesderivatives **4a**–**g** by the application of Suzuki cross coupling reactions [10]. To the best of our knowledge, there are only a few reports describing the *N*-acylation of sulfonamides under acidic condition [11] and Suzuki cross coupling reaction of 5-bromothiophene-acylsulfonamide (sulfacetamide). To carry out the synthesis, we started with the preparation of 5-bromothiophene-2-sulfonamide (**2**) by reaction of 2-bromo-thiophene with chlorosulfonic acid and PCl_5_, followed by the addition of ammonia as reported previously [12] (Scheme 1).

**Scheme 2:**
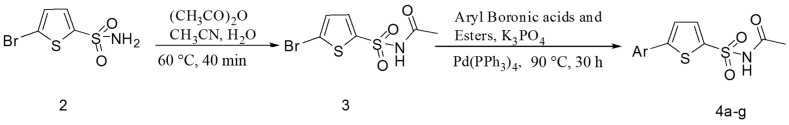
Synthesis of 5-bromothiophene-2-sulfonyl acetamide (**3**). *Reagents and conditions*: (i) **2** (0.002 mmol), acetic anhydride (0.0031 mmol), acetonitrile (5 mL); (ii) **3** (0.704 mmol), aryl boronic acids or arylboronic acid pinacol esters (0.774 mmol), K_3_PO_4_ (1.409 mmol), Pd (PPh_3_)_4_ (5 mol %), solvent/H_2_O (4:1), (see Table 1), 90 °C, 30 h.

Furthermore, we synthesized *N*-((5-bromothiophen-2-yl)sulfonyl)acetamide (sulfacetamide) (**3**, Scheme 2) by reacting **2** with acetic anhydride in acetonitrile in the presence of few drops of sulfuric acid by using method described by Martin *et al.* [1]. The Suzuki reaction of *N*-((5-bromothiophen-2-yl)sulfonyl)acetamide (**3**, 0.704 mmol) with 0.774 mmol of different aryl boronic acids and boronic esters gave 5-arylthiophene-2-sulfonylacetamides **4a**–**g** in moderate to very good yields (Table 1) [13].

**Table 1:** Synthesis of 5-arylthiophene-2-sulfonylacetamide **4a**–**g**.

Entry	Reagent	Product	Solvent/H_2_O (4:1)	Yields% ^a^
1	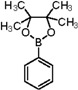	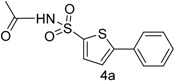	1,4-Dioxane	77
2	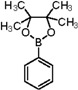	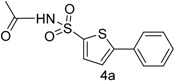	Toluene	68
3	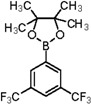	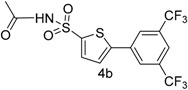	1,4-Dioxane	66
4	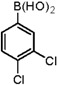	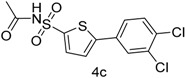	1,4-Dioxane	68
5	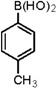	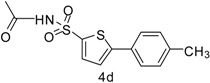	1,4-Dioxane	72
6	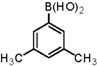	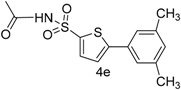	1,4-Dioxane	74
7	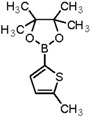	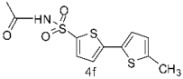	1,4-Dioxane	70
8	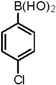	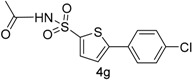	1,4-Dioxane	65

^a^ Isolated yield conditions: (95 °C, 30 h). The yields summarized in Table 1 are based on one time Suzuki cross coupling reaction.

In this context, we first focused our work on the optimization of the reaction conditions by studying the effect of solvent, base, catalyst and reflux time. It was noted that 5-bromothiophene-2-sulfonylacetamide (**3**, 0.704 mmol) reacted with phenyl boronic ester (0.774 mmol) in the presence of base K_3_PO_4_ (1.409 mmol), and Pd(PPh_3_)_4_ (5 mol %) in aqueous 1,4-dioxane (4:1 solvent/water ratio) to give a 77% yield of desired product **4a** after refluxing at 90 °C for 30 h (Table 1). Furthermore, the Pd[0] catalyzed reaction of *N*-((5-bromothiophen-2-yl)sulfonyl)acetamide (**3**, 0.704 mmol) with similar phenyl boronic esters (0.774 mmol) in toluene-water (4:1 solvent/water ratio) at 90 °C for 30 h in the presence of Pd (PPh_3_)_4_ (5 mol %) as a catalyst, to afford a moderate yield (64%, Table 1) of the desired product **4a**. It was noted that Pd[0] Suzuki cross coupling reactions of **3** with 3,5-methyl-ditrifluoromethylphenyl boronic ester, 3,4-dichlorophenyl boronic acid and 4-chlorophenyl boronic acid (0.774 mmol) afforded the desired compounds **4b**, **4c** and **4g** in 66%, 68% and 65% yields, respectively (Table 1). In contrast, we observed that the reaction of **3** with 4-methylphenyl boronic acid, 3,5-dimethyl-phenyl boronic acid and 5 methylthiopheneboronic in the presence of 1,4-dioxaneand K_3_PO_4_ (1.409 mmol) as a base and 5 mol % (tetrakis)Pd as catalyst successfully gave products **4d**, **4e** and **4f** in 72%, 74% and 70% yields, respectively (Table 1).

It was noted that the electron rich and electron poor aryboronic acids and esters had a great effect on the Pd[0] catalyzed Suzuki coupling reaction and the yields of the desired products. In these reactions, the base first converts the boronic acids and boronic esters into boronate ion, which is transmetallated faster than neutral boronic acid. The selectivity of this process is based on the fact that the boronate ion is a superior nucleophile and fastly replaces bromide ion in the Pd(II) (intermediate) complex [14]. For structure determination, the protons of the phenyl and thiophene rings of **4a**–**g** having electron donating and withdrawing functionality exhibited a chemical shift range of δ 7.90–6.97 ppm, while the protons of the amide functionality showed chemical shift values in range of δ 8.77–8.44 ppm. Further confirmation of the structures **4a**–**g** allowed assignment of the singlet peak appearing at δ 2.09–2.12 ppm to the 3H of the acetyl group, respectively. The ^1^H-NMR and mass spectra of compounds **4c** and **4e** are provided in the Supplementary Materials.

### 2.2. DFT Studies

#### 2.2.1. Geometry Optimization

Nowadays computational based methods have become very popular to investigate the structure-activity relationships of compounds and density functional theory (DFT) study is one of the most widely used computational methods due to its accuracy and less time consumption. The energy minima geometries of all products **4a**–**g** were optimized using the Gaussian 09 program at the B3LYP/6-31G (d, p) level of DFT. Optimized geometries were further used for structural investigations like frontier molecular orbitals analysis (FMOs), electrostatic potential (ESP) mapping and nonlinear optics (NLO) properties measurement.

#### 2.2.2. Frontier Molecular Orbital (FMO) Analysis

FMO analysis using computational methods is widely employed to explain the electronic as well as the optical properties of organic compounds [15]. During molecular interactions, the highest occupied molecular orbital (HOMO) and lowest unoccupied molecular orbital (LUMO) are the main participants. The HOMO donates electrons and its energy corresponds to the ionization potential (I.P.), whereas the LUMO accepts the electrons and its energy corresponds to the electron affinity (E.A.).

**Figure 1:**
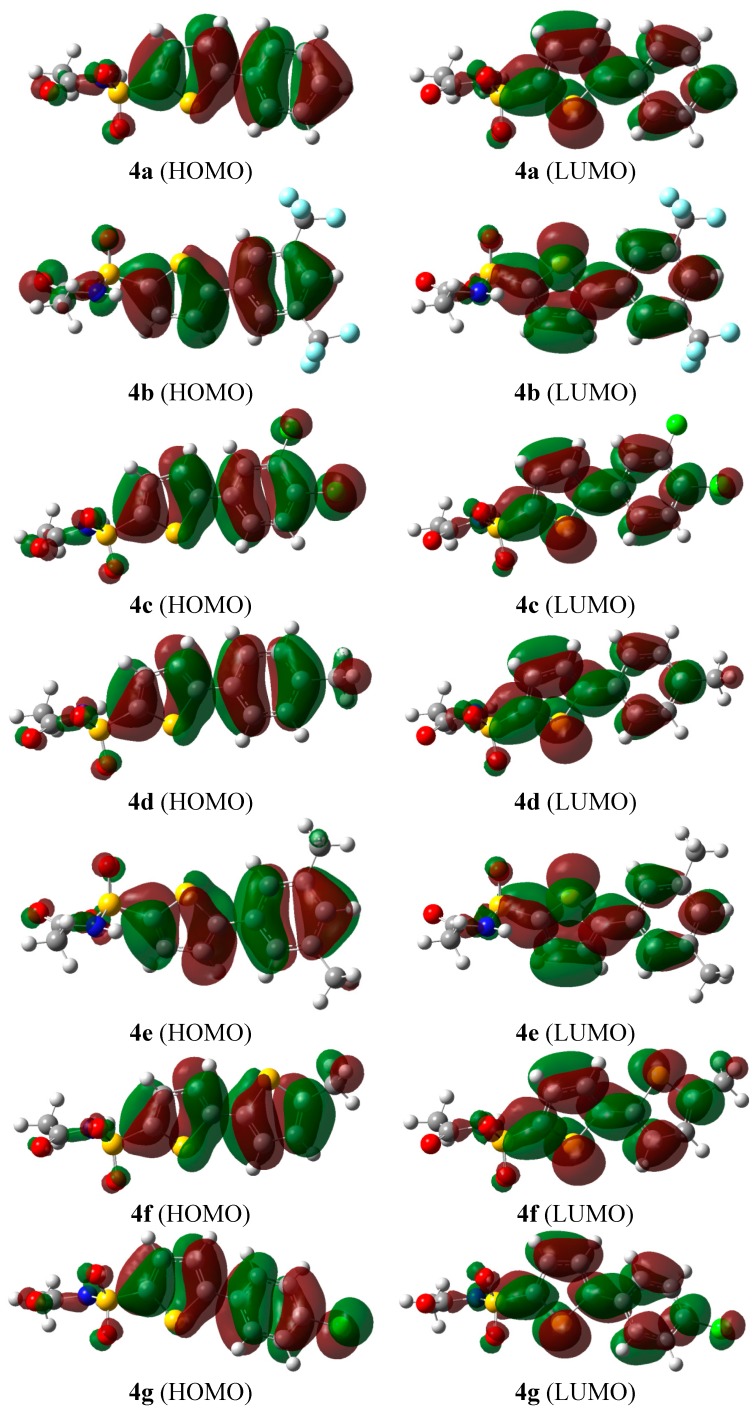
HOMO-LUMO surfaces of all products **4a**–**g**.

FMO analysis of all products **4a**–**g** was performed at the same level as used for optimization. The resulting HOMO-LUMO surfaces are shown in Figure 1, and their corresponding energies along with the energy gaps are listed in Table 2. The FMO analysis revealed that the isodensities are mainly concentrated on thiophene and aromatic moieties. The HOMO-LUMO energy gap for **4b** is found to be the highest and equal to 4.60 eV whereas the lowest band gap (3.99 eV) was observed for **4f**. The isodensity in the HOMO of **4b** is mainly spread only on the thiophene and phenyl rings, which reflects less conjugation in **4b**. This observation indicates the HOMO-LUMO gap in **4b** should display the highest energy among all products studied, and indeed this was the case. The high energy gap in **4b** would render it more stable towards ionization (*vide infra*).

**Table 2:** HOMO and LUMO energies along with HOMO-LUMO energy gap of products **4a**–**g**.

Entry	HOMO (a.u.)	LUMO (a.u.)	HOMO-LUMO (ΔE/eV)
**4a**	−0.24078	−0.07273	4.57
**4b**	−0.26085	−0.09138	4.60
**4c**	−0.24906	−0.08507	4.46
**4d**	−0.23451	−0.07014	4.47
**4e**	−0.23540	−0.06935	4.51
**4f**	−0.22386	−0.07710	3.99
**4g**	−0.24329	−0.07937	4.45

In **4a** the is density on the HOMO is more or less spread over the same region as compared to **4b**, therefore its energy gap is comparable to that of **4b** (*i.e.*, of 4.57 eV). On the other hand, the HOMO of **4f** has the maximum density spread over the entire scaffold (including the CH_3_ substituent, which results in the lowest energy gap (3.99 eV) and highest reactivity among all the compounds. The lowest energy gap in **4f** is expected due to the presence of planarity and the electron donating effect of the methyl group. The extent of conjugation in the LUMO in all compounds is almost similar (Figure 1), however, the intensities are slightly different.

#### 2.2.3. Molecular Electrostatic Potential (MEP)

One of the most interesting feature of quantum chemical investigations is to explain the reactivity of compounds under investigation. In term of reactivity, electrostatic potential also plays an important role in explaining reactivity. The reactivity of chemical systems can be explained by predicting electrophilic as well as nucleophilic sites in target molecules [16]. Mathematically, MEP can be expressed by using the following equation:

(1) $$V\left(r\right)=\sum^{\text{​}}\frac{Z_{A}}{\left|R_{A}-r\right|}-\int^{\text{​}}\frac{\text{ρ}\left(r^{\prime }\right)}{\left|r^{\prime }-r\right|}dr^{\prime }$$

**Figure 2:**
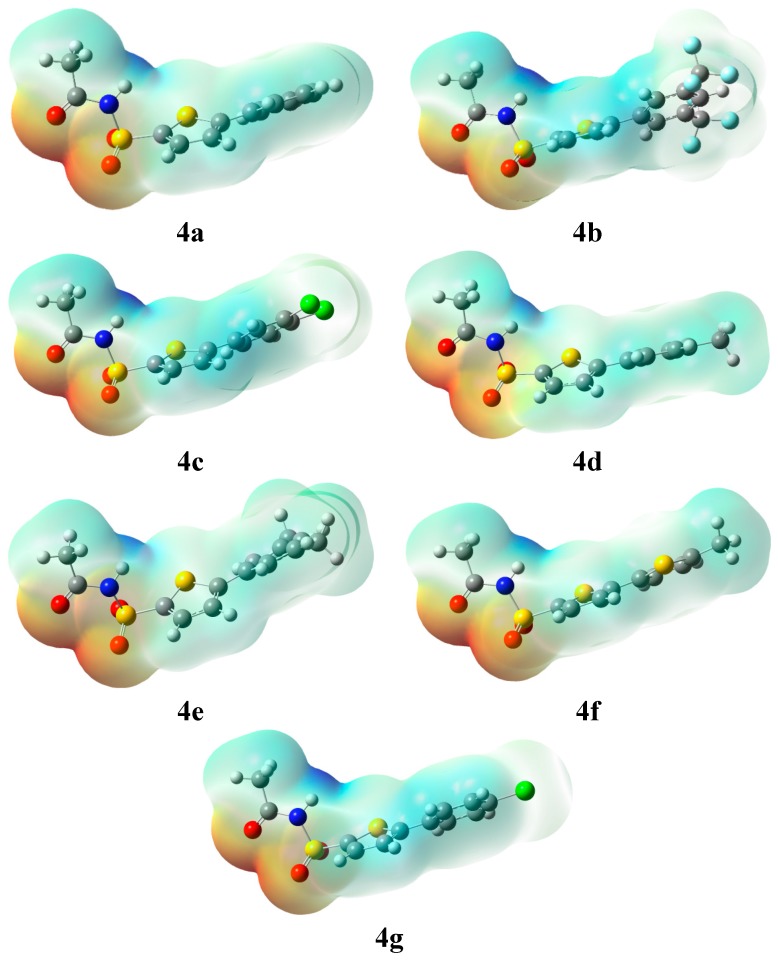
MEP Surfaces of all products **4a**–**g**.

Summation (∑) runs over all nuclei, *Z*_A_ is charge of nucleus, located at distance *R*_A_ and ρ(*r*′) is the electron density. MEP mapping using DFT methods can be useful in structural biology to determine ligand-substrate interactions, drug receptor and in enzyme-substrate interactions [17]. During MEP mapping two regions red and blue appear, the preferred nucleophilic site is represented by red color and the preferred electrophilic site is represented by blue color. The molecular electrostatic potential of all products **4a**–**g** was computed at the B3LYP/6-31G (d, p) level of DFT and the surfaces are shown in Figure 2.

MEP analysis of all products **4a**–**g** revealed that the negative potential is concentrated on the oxygen of the sulfacetamide moiety (attached to the thiophene ring) and as a result this is the preferred site for electrophilic attack as well as for cations, whereas a positive potential indicating the site for nucleophilic attack is concentrated on the NH group of the sulfacetamide. The negative and positive potential values of individual products are listed in Table 3.

**Table 3:** Values of −ve and +ve potential of products **4a**–**g**, computed at the DFT/B3LYP/6-31G (d, p) level.

Entry	−ve Potential (a.u.)	+ve Potential (a.u.)
**4a**	−0.07342	0.07342
**4b**	−0.07182	0.07182
**4c**	−0.06974	0.06974
**4d**	−0.07444	0.07014
**4e**	−0.07459	0.07459
**4f**	−0.07400	0.07400
**4g**	−0.07096	0.07096

From the results in Table 3, it is clear that the −ve and +ve potential of all products are found to be almost in the same range and no significant difference was observed. The smallest value was observed for **4c**, which ranged from −0.06974 a.u. to 0.06974 a.u. (−ve sign is showing the −ve potential and *vice versa*), whereas the highest value was observed for **4e**, (−0.07459 a.u. to 0.07459 a.u.).

#### 2.2.4. Hyperpolarizability and Non-Linear Optical (NLO) Properties

Materials having high nonlinear optics (NLO) responses are very useful in optoelectronic devices, and non-linear optics have great applications in information technology, and in other industries [18]. NLO materials also have wide range applications for photonic communication and digital memory devices, national defense and the pharmaceutical industry [19]. Compounds having electron donor and electron acceptor groups along with a π-conjugated system can be considered as strong candidates for such applications. In order to investigate the relation between molecular structure and NLO properties, the first hyperpolarizibility value of all compounds **4a**–**g** was simulated at the B3LYP/6-31G (d, p) level of theory along with the additional keyword POLAR and mathematically calculated using the following equation:

β _tot_= [(β_xxx_+ β_xyy_+ β_xzz_) ^2^+ (β_yyy_+ β_yzz_+ β_yxx_) ^2^+ (β_zzz_+ β_zxx_+ β_zyy_) ^2^]^1/2^ (2)

The value of the first hyperpolarazibility is determined in a.u. and then converted to esu using the conversion factor 1 a.u. = 8.6393 × 10^−33^ esu. The calculated first hyperpolarizibility parameters of compounds **4a**–**g** are given in Table 4. As reflected from the data, the first hyperpolarizibility values show the same trend according to the electron donating and withdrawing capacity, and extended π-conjugation pattern. Compound **4b** has the lowest value of hyperpolarazibility (1.857 × 10^−30^ esu), because of the similar electronic nature at both termini. On the other hand **4f**, having a CH_3_ group on one side (electron donating) and a sulfonamide (electron withdrawing) on the other showed the highest value (12.879 × 10^−30^ esu), which suggests that **4f** has the potential to serve as a candidate for non-linear optical material. Furthermore, the first hyperpolarizability can also be correlated to the HOMO-LUMO energy gap. Compound **4b** (with the highest energy gap, 4.60 eV) has the lowest hyper-polarizability value. On the other hand, the easy flow of electrons in **4f** from one terminus of the molecule to the other renders a higher hyperpolarizability value, and **4f** also has lowest band gap (HOMO-LUMO) being the most reactive among all the products as well.

**Table 4:** First hyperpolarizability parameters of **4a**–**g**.

Entry	4a	4b	4c	4d	4e	4f	4g
β_xxx_	−546.18	118.57	1043.62	−1159.69	−804.94	1627.21	1301.99
β_xxy_	−27.13	66.68	−16.19	−94.92	56.93	−184.46	−122.23
β_xyy_	26.92	53.51	10.45	45.99	22.73	−31.44	11.45
β_yyy_	32.77	−49.74	−60.06	26.81	−67.96	44.77	23.43
β_xxz_	−37.90	59.80	−83.06	−18.56	−47.42	−66.36	−2.37
Β_xyz_	6.16	5.61	−5.87	0.29	13.17	2.75	4.35
β_yyz_	1.38	−9.85	1.38	1.27	−10.85	−25.01	4.56
β_xzz_	95.61	33.85	−19.52	109.00	58.63	−112.32	80.98
β_yzz_	18.20	−1.45	8.08	25.42	3.92	19.57	2.39
β_zzz_	33.17	10.04	19.56	41.49	56.31	4.35	51.02
β_tot_ × 10^−33^ (esu)	3.67	1.857	8.973	8.690	6.251	12.879	11.998

### 2.3. Biological Activity

#### 2.3.1. Urease Inhibition Activity

Urease is an enzyme which converts urea to ammonium carbonate and is used as a diagnostic tool to establish the presence of different pathogens in the urinary and gastrointestinal tract [20]. The percentage activity values of urease enzyme of compounds **4a**–**d** were measured at 50 µg/mL and 250 µg/mL (Table 5), whereas, for compounds **4e**–**g**, the percentage activity values of urease enzyme were measured at 15, 40 and 80 µg/mL, respectively (Table 6). Thiourea that showed IC_50_ values of 43 µg/mL and 23.3 µg/mL at various concentrations was used as a standard drug.

**Table 5:** Urease inhibition studies of 5-arylthiophene-2-sulfonylacetamides **4a**–**d**.

Entry	Percentage Activity at 50 µg/mL	Percentage Activity at 250 µg/mL	IC_50_ µg/mL
**4a**	67.56 ± 0.007	89 ± 0.01	38.4 ± 0.32
**4b**	29.98 ± 0.034	56 ± 0.006	82.01 ± 0.79
**4c**	54.4 ± 0.002	78 ± 0.003	42.5 ± 0.41
**4d**	13.34 ± 0.007	54 ± 0.004	218 ± 1.98
Standard	60 ± 0.032	95 ± 0.09	43 ± 0.38

**Table 6:** Urease inhibition studies of 5-arylthiophene-2-sulfonylacetamides **4e**–**g**.

Entry	Percentage Activity at 15 µg/mL	Percentage Activity at 40 µg/mL	Percentage Activity at 80 µg/mL	IC_50_ µg/mL
**4e**	44.59 ± 0.14	91.21 ± 0.81	92.88 ± 0.14	17.9 ± 0.13
**4f**	42.44 ± 0.11	92.12 ± 0.21	94.66 ± 0.11	17.1 ± 0.15
**4g**	46.23 ± 0.11	90.97 ± 0.18	68 ± 0.02	23.3 ± 0.21
Standard	47.1 ± 0.31	65 ± 0.01		

Urease inhibition activity of this newly synthesized 5-arylthiophene-2-sulfonylacetamide compounds **4a**–**g** was investigated to gain knowledge about their possible interaction with the active site of the enzyme. Since Urease belongs to the family of hydrolases any restrain in the activity of the enzyme would be expected to express in terms of reduced hydrolysis of its substrate [21]. Herein, we show that the compound **4f**, *N*-((5′-methyl-[2,2′-bithiophen]-5-yl)sulfonyl)acetamide, showed excellent urease inhibition activity at 40 µg/mL and 80 µg/mL concentrations where the percentage inhibition values were found to be 92.12 ± 0.21 and 94.66 ± 0.11, respectively with an IC_50_ value ~17.1 ± 0.15 µg/mL. This was followed by compounds **4e**, *N*-((5-(3,5-dimethylphenyl)thiophen-2-yl)sulfonyl)acetamide, and **4g**, *N*-((5-(4-chlorophenyl)thiophen-2-yl)sulfonyl)acetamide, that showed significant urease inhibition with IC_50_ values of 17.9 + 0.13 and 23.3 + 0.21 µg/mL, respectively. Compound **4a**, *N*-((5-phenylthiophen-2-yl)sulfonyl)acetamide showed good urease inhibition at 50 µg/mL and 250 µg/mL concentrations, where the percentage inhibition values were found to be 67.56 ± 0.007 and 89 ± 0.01, respectively, in addition to an IC_50_ value ~38.4 µg/mL. *N*-((5-(*p*-Tolyl)thiophen-2-yl)sulfonyl)acetamide (**4d**) showed a 13.34 ± 0.007 value of percentage inhibition at 50 µg/mL concentration along with an IC_50_ value ~43 µg/mL. For the compounds *N*-((5-(3,5-bis(trifluoromethyl)phenyl)thiophen-2-yl)sulfonyl)acetamide (**4b**) and *N*-((5-(3,4-dichlorophenyl)thiophen-2-yl)sulfonyl)acetamide (**4c**) moderate urease inhibition activities were observed, with percentage inhibition values ~56 ± 0.006 and 78.11 ± 0.003 at 250 µg/mL concentration, along with IC_50_ values ~82.01 µg/mL and 42.5 µg/mL, respectively (Table 6), suggesting that the electronic effects and presence of different functional groups on aromatic rings and sulfacetamide moiety had a strong effect on the urease inhibitory action of these compounds. Since, the electronic and steric factors have great influence on the biological activities [22], it was surprising to see that the electron withdrawing and electron donating functional groups present on the benzene ring exhibited low and high inhibitor action against urease enzyme [23]. Note worthily, electron withdrawing groups decrease the metal chelating activity and *vice versa*, therefore, the removal of Ni^2+^ ions through chelation may result in the inactivation of the enzyme. It is further concluded that the urease inhibitory activity might be affected by the presence of the electronic effects of functional groups and the position of functional groups in the **4a**–**g** series of compounds.

#### 2.3.2. Antibacterial Activity

Kulsoom *et al.* reported that sulfacetamides are effective against different *Escherichia coli* and *Staphylococcus aureus* strains and sulfacetamide suspension can be used for the treatment of eye infections caused by *Staphylococcus aureus* [24]. Therefore, we examined all newly synthesized compounds **4a**–**g** for their antibacterial activity. A general antibacterial sensitivity test (inhibition zone, mm) was performed on six test organisms (*Bacillus subtiles*, *Escherichia coli*, *Staphylococcus aureus*, *Shigella dysenteriae*, *Salmonella typhae*, *Pseudomonas aeruginosa*) by using agar diffusion according to the reported methods [25] for all the new 5-bromothiophene-2-sulfonylacetamide derivatives and the clinical standard ampicillin. The antibacterial activity of all new 5-arylthiophene-2-sulfonylacetamide (sulfacetamide) derivatives **4a**–**g** was authenticated via *in vitro* screening against six strains and was calculated from zones of inhibition (ZOI). The obtained antibacterial results at different concentrations (100, 300 and 1000 µg) are summarized in Table 7, Table 8 and Table 9, respectively. *N*-((5-Phenylthiophen-2-yl)sulfonyl)acetamide (**4a**) showed the highest activity against *Shigella dysenteriae* at 100 µg and 1000 µg concentration with percentage activity values ~55.7 ± 0.016 and 80 ± 0.00, respectively (Table 7 and Table 9). Noticeably, *N*-((5-(3,5-bis(trifluoromethyl)-phenyl)thiophen-2-yl)sulfonyl)acetamide (**4b**) produced the highest activity against *Escherichia coli* at 100 µg and 1000 µg concentrations, with percentage activity values ~20 ± 0.01 and 62 ± 0.004, respectively. At 300 µg concentration, compound **4b** also showed the highest activity against *Pseudomonas aeruginosa* with a percentage activity value ~44.81 ± 0.009 as shown in Table 8. Note worthily, *N*-((5-(3,4-dichlorophenyl)thiophen-2-yl)sulfonyl)acetamide (**4c**) exhibited a high percentage of activity against a *Salmonella typhae* stain at 1000 µg with a value ~64 ± 0.0012 (Table 9). It was found that the compound *N*-((5-(4-chlorophenyl)thiophen-2-yl)sulfonyl)acetamide (**4g**) exhibited the highest activity against *Bacillus subtiles* with percentage activity value ~78 ± 0.007 at 1000 µg concentration. *N*-((5-(*p*-tolyl)thiophen-2-yl)sulfonyl)acetamide (**4d**) also showed a promising percentage of antibacterial activity against a *Shigella dysenteriae* strain, with a value ~52.36 ± 0.002 at 300 µg. The present study also examined the response of *N*-((5-(3,5-dimethylphenyl)thiophen-2-yl)sulfonyl)acetamide (**4e**) against *Pseudomonas aeruginosa* at 1000 µg concentration with an observed percentage activity value ~67 ± 0.04. *N*-((5′-methyl-[2,2′-bithiophen]-5-yl)sulfonyl)acetamide (**4f**) also showed noticeable bacterial inhibition, as can be seen from the values outlined in Table 7.

Taken together, it is very significant to note that all newly synthesized 5-arylthiophene-2-sulfonylacetamide derivatives of gave better percentage antibacterial activity results ranging from 100 µg to 1000 µg concentrations. In addition, all compounds showed a concentration dependent inhibitory effect in the *in vitro* microbial growth assay [26]. It is worth considering that the antibacterial activity in all these compounds may depend on the nature of the substituent (R) group, even though all the compounds showed significant antibacterial inhibition. It was concluded that sulfacetamide derivatives with electron donating and electron withdrawing functional groups may lead to a much stronger and more effective antibacterial activity.

**Table 7:** Antibacterial activities (100 µg) of 5-arylthiophene-2-sulfonylacetamide **4a**–**g**.

% Activity at 100 µg						
Entry	Gram Positive Bacteria	Gram Negative Bacteria	Gram Positive Bacteria	Gram Negative Bacteria	Gram Negative Bacteria	Gram Negative Bacteria
	*Bacillus subtiles*	*Escherichia coli*	*Staphylococcus aureus*	*Shigella dysenteriae*	*Salmonella typhae*	*Pseudomonas aeruginosa*
**4a**	17 ± 0.0007	13 ± 0.007	19.03 ± 0.0	55.7 ± 0.016	39.31 ± 0.008	39.32 ± 0.004
**4b**	17 ± 0.005	20 ± 0.01	17.7 ± 0.007	38.59 ± 0.0007	36.57 ± 0.005	32.0 ± 0.006
**4c**	14.1 ± 0.002	16 ± 0.00	13.89 ± 0.002	15.59 ± 0.004	34.95 ± 0.012	27.2 ± 0.004
**4d**	20 ± 0.007	20 ± 0.007	18.35 ± 0.0007	19.93 ± 0.038	33.26 ± 0.002	24.1 ± 0.004
**4e**	15 ± 0.035	20 ± 0.035	17.96 ± 0.0007	26.93 ± 0.038	33.70 ± 0.009	25.05 ± 0.005
**4f**	19 ± 0.007	12 ± 0.001	19.86 ± 0.001	38.33 ± 0.0007	48.55 ± 0.014	35.05 ± 0.004
**4g**	22 ± 0.019	12. ± 0.01	20.17 ± 0.02	31.1 ± 0.009	32.51 ± 0.006	32.06 ± 0.004
Ampicillin	60.2 ± 0.32	82 ± 0.2	65 ± 0.22	60 ± 0.18	86 ± 0.5	55 ± 0.12

**Table 8:** Antibacterial activities (300 µg) of 5-arylthiophene-2-sulfonylacetamide **4a**–**g**.

% Activity at 300 µg						
Entry	Gram Positive Bacteria	Gram Negative Bacteria	Gram Positive Bacteria	Gram Negative Bacteria	Gram Negative Bacteria	Gram Negative Bacteria
	*Bacillus subtiles*	*Escherichia coli*	*Staphylococcus aureus*	*Shigella dysenteriae*	*Salmonella typhae*	*Pseudomonas aeruginosa*
**4a**	34 ± 0.06	29 ± 0.06	32.22 ± 0.002	51.78 ± 0.001	29.06 ± 0.016	38.53 ± 0.010
**4b**	28.2 ± 0.01	30 ± 0.02	32.16 ± 0.0007	51.7 ± 0.000	36.12 ± 0.006	44.81 ± 0.009
**4c**	26.3 ± 0.001	27 ± 0.006	22.50 ± 0.003	50.17 ± 0.001	31.2 ± 0.0091	40.64 ± 0.03
**4d**	40.0 ± 0.000	34 ± 0.05	27.35 ± 0.0003	52.36 ± 0.002	34.22 ± 0.0007	40.05 ± 0.038
**4e**	40.01 ± 0.001	40 ± 0.01	27.80 ± 0.0004	45.96 ± 0.017	28.8 ± 0.021	40.74 ± 0.06
**4f**	24.5 ± 0.00	25 ± 0.00	28.74 ± 0.0007	49.30 ± 0.023	33.58 ± 0.007	41.86 ± 0.043
**4g**	37 ± 0.144	35 ± 0.144	29.57 ± 0.0007	52.94 ± 0.001	40.2 ± 0.016	38.4 ± 0.003
Ampicillin	85 ± 0.51	88 ± 0.6	78 ± 0.45	76.2 ± 0.29	89 ± 0.18	72 ± 0.61

**Table 9:** Antibacterial activities (1000 µg) of 5-arylthiophene-2-sulfonylacetamide **4a**–**g**.

% Activity at 1000 µg						
Entry	Gram Positive Bacteria	Gram Negative Bacteria	Gram Positive Bacteria	Gram Negative Bacteria	Gram Negative Bacteria	Gram Negative Bacteria
	*Bacillus subtiles*	*Escherichia coli*	*Staphylococcus aureus*	*Shigella dysenteriae*	*Salmonella typhae*	*Pseudomonas aeruginosa*
**4a**	60 ± 0.004	64 ± 0.004	64 ± 0.004	80 ± 0.00	55 ± 0.009	46 ± 0.12
**4b**	32 ± 0.004	62 ± 0.004	59 ± 0.0021	73 ± 0.0034	56 ± 0.007	45 ± 0.0021
**4c**	31 ± 0.0003	44 ± 0.001	58 ± 0.005	74 ± 0.015	64 ± 0.0012	48 ± 0.045
**4d**	54 ± 0.009	59 ± 0.01	59 ± 0.00	75 ± 0.023	62 ± 0.003	65 ± 0.03
**4e**	75 ± 0.01	74 ± 0.01	55.5 ± 0.0012	65 ± 0.004	58 ± 0.0054	67 ± 0.04
**4f**	34 ± 0.0021	54 ± 0.00	56 ± 0.004	67 ± 0.045	65 ± 0.001	64 ± 0.0012
**4g**	78 ± 0.007	71 ± 0.007	61 ± 0.006	75 ± 0.02	68 ± 0.0004	43 ± 0.5
Ampicillin	92 ± 0.55	95.9 ± 0.21	92.3 ± 0.32	91.6 ± 0.61	98.9 ± 0.26	92 ± 0.44

## 3. Experimental Section

### 3.1. General Information

All reagents and chemicals were purchased from Sigma Aldrich (St. Louis, MO, USA) and Alfa Aesar (Heysham, Lancashire, UK). A B-540 melting point apparatus (Büchi Labortechnik AG, Switzerland) was used to record the melting points. ^1^H-NMR and ^13^C-NMR spectrum was measured in CDCl_3_ and CD_3_OD at 400/100 MHz on an Aspect AM-400 instrument (Bruker, Billerica, MI, USA). Chemical shifts are reported in δ (ppm) units, while the coupling constant values were reported in Hz. A Jeol JMS 600 mass spectrometers with a data system (Akishima, Tokyo, Japan) was used to record EI-MS spectrum. Column chromatography was used to purify compounds, using silica gel (230–400 mesh size and 70–230 mesh size). Silica gel 60 PF_254_ TLC plates (Merck, Germany) were used to monitor all reactions. The newly synthesized compounds were detected/visualized by UV (254–365 nm).

### 3.2. Synthesis of 5-Bromothiophene-2-sulphonamide (**2**)

For the synthesis of 5-bromothiophene-2-sulfonamide from 2-bromothiophene (**1**), freshly distilled chlorosulfonic acid solution was added drop wise under continuous stirring to a solution of compound **1** (12 mmol) in CCl_4_ (6 mL), cooled to −30 °C. Stirring was done for another 30 min at −25 °C, and the mixture was then kept at room temperature for another 30 min. The organic layer was separated and solvent was removed under reduced pressure. Next, 25% ammonia solution (50 mL) was mixed with the residue, stirred for 3 h and neutralized with 10% HCl. The desired product was filtered, dried and compared with previously reported 5-bromothiophene-2-sulphonamide [12].

### 3.3. Synthesis of 5-Bromothiophene-2-sulfonylacetamide (Sulfacetamide) (**3**)

To an acetonitrile solution (5 mL) containing 5-bromothiophene-2-sulfonamide (**2**, 0.002 mmol) and acetic anhydride (0.0031 mmol), a few drops of concentrated sulfuric acid were added and the mixture was stirred for 40 min at 60 °C under a nitrogen atmosphere. Later, distilled water (about 15–20 mL) was added to this solution and stirred for 1 h at room temperature. The solution was then filtered and the precipitates were collected, washed and dried. Further purification and identification was done by flash chromatography and various spectroscopic techniques, respectively [1]. M.P. 118–120 °C; ^1^H-NMR (CDCl_3_): δ 8.44 (s, 1H, NH), 7.62 (d, *J* = 4.4, 1H-thiophene), 7.09 ( d, *J* = 4, 1H-thiophene), 2.12 (s, 3H, CH_3_), ^13^C-NMR (CDCl_3_ + CD_3_OD): δ = 23.6, 122.2, 130.2, 135.4, 174.2; EI-MS (*m*/*z*, + ionmode): 284.00 [M + H]^+^; 282.00; [M − SO_2_]^+^ = 219; [ M − NH and acetyl fragment]^+^ = 226; [M − SO_2_ and acetyl fragment]^+^ = 178. Anal. Calcd. For C_6_H_6_NBrO_3_S_2_: C, 25.36; H, 2.13; N, 4.93; Found: C, 25.46; H, 2.52; N, 5.12.

### 3.4. General Procedure for the Synthesis of 5-Arylthiophene-2-sulfonylacetamides **4a**–**g**

To a 1,4-dioxane (3 mL) solution of 5-bromothiophene-2-sulfonylacetamide (**3**, 0.704 mmol) 5 mol % Pd(PPh_3_)_4_ was added and the resulting mixture stirred for 30 min at room temperature under a nitrogen atmosphere. Next, arylboronic acids and arylboronic esters (0.774 mmol), and potassium phosphate (1.409 mmol) were added along with water (1.5 mL) under a nitrogen atmosphere. The solution was stirred at 95 °C for 30 h and later cooled to 20 °C. Later on H_2_O was added and the reaction mixture was extracted with ethyl acetate to obtain an organic layer that was filtered and dried by the addition of MgSO_4_. The solvent was removed under reduced pressure. The residue was purified by column chromatography using ethyl acetate and *n*-hexane (1:1) to obtain the desired products which were characterized by spectroscopic techniques.

*N-((5-Phenylthiophen-2-yl)sulfonyl)acetamide* (**4a**). M.P. 158–160 °C; ^1^H-NMR (CDCl_3_): δ 8.77 (s, 1H, NH), 7.61–7.00 (m, 5H-Ar, 2H-thiophene), 2.10 (s, 3H, CH_3_). ^13^C-NMR (CDCl_3_ + CD_3_OD): δ = 23.6, 127.7, 128.2, 129.1, 129.9, 130.2, 132.1, 135.3, 139.7, 174.1; EI-MS (*m*/*z*, + ionmode): 281.00 [M + H]^+^; 282.00. Anal. Calcd. For C_12_H_11_NO_3_S_2_: C, 51.23; H, 3.94; N, 4.98. Found: C, 51.28; H, 3.96; N, 4.98.

*N-((5-(3,5-Bis(trifluoromethyl)phenyl)thiophen-2-yl)sulfonyl)acetamide* (**4b**). M.P. 168 °C; ^1^H-NMR (CDCl_3_): δ 8.77 (s, 1H, NH), 7.9–7.10 (m, 3H-Ar, 2H-thiophene), 2.12 (s, 3H, CH_3_). ^13^C-NMR (CDCl_3_ + CD_3_OD): 24.0, 123.3, 125.1, 127.3, 129.2, 130.4, 133.2, 134.6, 136.4, 139.8, 174.3; EI-MS (*m*/*z*, −ionmode): 416.08 [M − H]^+^. Anal. Calcd. For C1_4_H_9_F_6_NO_3_S_2_: C, 40.29; H, 2.17; N, 3.36. Found: C, 40.46; H, 2.52; N, 3.42.

*N-((5-(3,4-Dichlorophenyl)thiophen-2-yl)sulfonyl)acetamide* (**4c**). M.P. 148–150 °C; ^1^H-NMR (CDCl_3_): δ 8.77 (s, 1H, NH), 7.30–7.85 (m, 4H-Ar, 2H-thiophene), 2.10 (s, 3H, CH_3_). ^13^C-NMR (CDCl_3_ + CD_3_OD): δ = 23.8, 123.8, 128.1, 129.2, 130.1, 131.4, 132.0, 133.4, 133.2, 139.0, 173.8; EI-MS (*m*/*z*, +ionmode): 350.00 [M + H]^+^; 351.17; [M – O]^+^ = 336.50; [M − NH and acetyl fragment and SO_2_]^+^ = 273. Anal. Calcd. For C_12_H_9_Cl_2_NO_3_S_2_: C, 41.15; H, 2.59; N, 4.00. Found: C, 41.59; H, 2.60; N, 4.02.

*N-((5-(p-Tolyl)thiophen-2-yl)sulfonyl)acetamide* (**4d**). M.P. 161–163 °C; ^1^H-NMR (CDCl_3_): δ 8.77 (s, 1H, NH), 7.62–7.09 (m, 4H-Ar, 2H-thiophene), 2.09 (s, 3H, CH_3_), 2.34 (s, 3H, CH_3_). ^13^C-NMR (CDCl_3_ + CD_3_OD): δ = 23.8, 24.6, 127.8, 127.2, 128.6, 131.0, 132.2, 134.4, 134.7, 139.4, 173.4; EI-MS (*m*/*z*, −ionmode): 295.00 [M − H]^+^; 294.17. Anal. Calcd. For C_13_H_13_NO_3_S_2_: C, 52.86; H, 4.44; N, 4.74. Found: C, 52.88; H, 4.46; N, 4.76.

*N-((5-(3,5-Dimethylphenyl)thiophen-2-yl)sulfonyl)acetamide* (**4e**). M.P. 148–149 °C; ^1^H-NMR (CDCl_3_): δ 8.77 (s, 1H-NH), 7.65–6.97 (m, 3H-Ar, 2H-thiophene), 2.10 (s, 3H, CH_3_), 2.37 (s, 3H, 2CH_3_). ^13^C-NMR (CDCl_3_ + CD_3_OD): δ = 22.1, 23.4, 127.0, 127.9, 129.9, 131.0, 131.6, 134.2, 139.0, 139.3, 173.2; EI-MS (*m*/*z*, +ionmode): 309.40.00 [M − H]^+^; 308.25. Anal. Calcd. For C_14_H_15_NO_3_S_2_: C, 54.35; H, 4.89; N, 4.53. Found: C, 54.36; H, 4.52; N, 4.54.

*N-((5′-Methyl-[2,2′-bithiophen]-5-yl)sulfonyl)acetamide* (**4f**). M.P. 167–168.4 °C; ^1^H-NMR (CDCl_3_): δ 8.77 (s, 1H, NH), 7.71–7.09 (m, 3H-thiophene), 7.0 (d, *J* = 4.2, 1H-thiophene), 2.09 (s, 3H, CH_3_), 2.40 (s, 3H, CH_3_).^13^C-NMR (CDCl_3_ + CD_3_OD) :δ = 16.1, 23.6, 125.6, 128.0, 128.4, 129.2, 133.3, 137.6, 137.2, 139.3, 173.9; EI-MS (*m*/*z*, +ionmode): 301.00 [M + H]^+^; 302.04. Anal. Calcd. For C_11_H_11_NO_3_S_3_: C, 43.83; H, 3.68; N, 4.65. Found: C, 43.88; H, 3.68; N, 4.66.

*N-((5-(4-Chlorophenyl)thiophen-2-yl)sulfonyl)acetamide* (**4g**). M.P. 167–170 °C; ^1^H-NMR (CDCl_3_): δ 8.70 (s, 1H, NH), 7.75–7.08 (m, 4H-Ar, 2H-thiophene), 2.09 (s, 3H, CH_3_). ^13^C-NMR (CDCl_3_ + CD_3_OD): δ = 22.7, 128.8, 129.0, 129.9, 131.0, 132.2, 134.4, 134.7, 139.4, 173.4; EI-MS (*m*/*z*, +ionmode): 315.00 [M + H]^+^; 316.10. Anal. Calcd. For C_12_H_10_ClNO_3_S_2_: C, 45.64; H, 3.19; N, 4.44. Found: C, 45.66; H, 3.22; N, 4.48.

### 3.5. Computational Methods

Computational simulations of all products **4a**–**g** were performed using the Gaussian 09 software at density functional theory (DFT) level, as instituted in [27]. The visualization of the results and graphics was achieved using Gauss View 05 [28]. Energy minima optimization was carried out using the DFT/B3LYP/6-31G (d, p) basis set. Frequency simulations were performed at the same level, to confirm the optimized geometries as true energy minima (no imaginary frequency was observed). Furthermore optimized geometries were used for frontier molecular orbital (FMO) analysis, and molecular electrostatic potential (MEP) mapping. Nonlinear optics properties were computed at the same level of theory as used for optimization and just with the additional keyword POLAR.

### 3.6. Urease Inhibition Activity

Jack bean urease enzyme (25 µL) was added in buffer solution (55 µL) containing 100 mM urea to prepare the stock solution. This mixture was then incubated with 5 µL (0.5 mM concentration) of the newly synthesized compounds for 15 min at 30 °C in 96-well plates. Anti-urease activity was resolved by knowing the production of ammonia using the indophenol method [29]. Concisely, in each well, phenol reagent (45 µL, 0.005% *w*/*v* sodium nitroprusside and 1% *w*/*v* phenol) was added in addition to the alkali reagent (70 µL, NaOH 0.5% *w*/*v* and 0.1% NaOCl). After 50 min, the increase in absorbance was measured at 630 nm using a micro plate reader. All reactions were carried out in triplicate to obtained 200 µL final volume. The Softmax pro software was used to obtain the results as change in absorbance per minute. All assays were carried out at a specific pH of 6.8. The following formula was used to calculate the % inhibition:

100 − (OD test well/OD control) × 100(3)

Thiourea was used as standard inhibitor of urease [20]. The EZ-fit kinetic data base was used to determine IC_50_ values [30]. In the case of colored compounds, sample blanks were also prepared. Absorbance of sample blanks was subtracted from the absorbance of samples to get the corrected absorbance of the samples. Corrected absorbance of sample was used to calculate % age inhibition.

### 3.7. Antibacterial Activity Assay

#### Determination of Minimum Inhibitory Concentrations

All the sulfonamide derivatives were dissolved at 250 µg/mL concentration in dimethyl sulphoxide. Nutrient ager was composed of NaCl (10 g), bactotryptone (10 g) and (5 g) yeast extract with a final pH value of 7.4. After that, the mixture was left for 18 h to grow the six bacteria and after 18 h at 37 °C, nutrient broth was diluted in sterile broth. To achieve a final bacterial count of 1 × 10^6^ cell/mL, 1 mL from each dilution was added to 100 mL of cooled and sterilized nutrient agar media. These plates were kept at room temperature and dried at 37 °C for 20 h. Whatman No. 41 paper was used as paper discs for assays. Discs were soaked in test solutions of different concentrations and placed at regular intervals of 6–7 cm on inoculated agar media; there should not be any extra solution on the discs so care was taken when discs were soaked in solution. These plates were incubated at 37 °C, and antibacterial activity was determined by measuring the zone of inhibition in mm. Growth inhibition was calculated by the method of difference to positive control.

## 4. Conclusions

In the present study, we have reported the synthesis of 5-aryl thiophenes sulphonylacetamide (sulfacetamide) derivatives through Pd[0] catalyzed Suzuki cross coupling reactions of 5-bromothiophene acylsulfonamide with various aryl boronic acids/esters under mild conditions. A wide range of spectroscopic techniques, including ^1^H-NMR, ^13^C-NMR and mass spectroscopy were applied to elucidate the structure of the synthesized compounds. DFT investigations were performed to gain insight into the structure activity relationships. FMO analysis revealed that **4b** has the highest HOMO-LUMO energy gap (4.60 eV) and therefore is the least reactive among all the prepared derivatives, while as **4f** has lowest energy gap (3.99 eV) and was the most reactive. MEP mapping indicated the sites for nucleophilic as well as electrophilic attack over the entire geometry. First hyperpolarizability analysis revealed that **4f** has the highest value (12.879 × 10^−30^ esu) among all products, therefore it can act as a potential candidate for nonlinear optics applications. In addition, the synthesized compounds were explored as anti-urease and antibacterial molecules. This studied offered a preliminary structure-activity study of 5-aryl thiophenes bearing sulphonylacetamide moieties that have a great scope and potential use in pharmaceutical chemistry.

## Supplementary Materials

Supplementary materials can be accessed at: http://www.mdpi.com/1420-3049/20/11/19661/s1.
