# Supplementary material for: Synthesis, Density Functional Theory (DFT), Urease Inhibition and Antimicrobial Activities of 5-Aryl Thiophenes Bearing Sulphonylacetamide Moieties
Source: Molecules. 2015 Nov 5;20(11):19914–28. doi: 10.3390/molecules201119661 (PMC6332040; doi:10.3390/molecules201119661)
Supplement: Supplementary file 1 [file molecules-20-19661-s001.pdf]

molecules

MDPI

Supplementary Informations

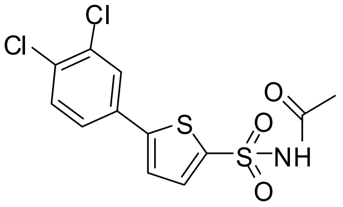

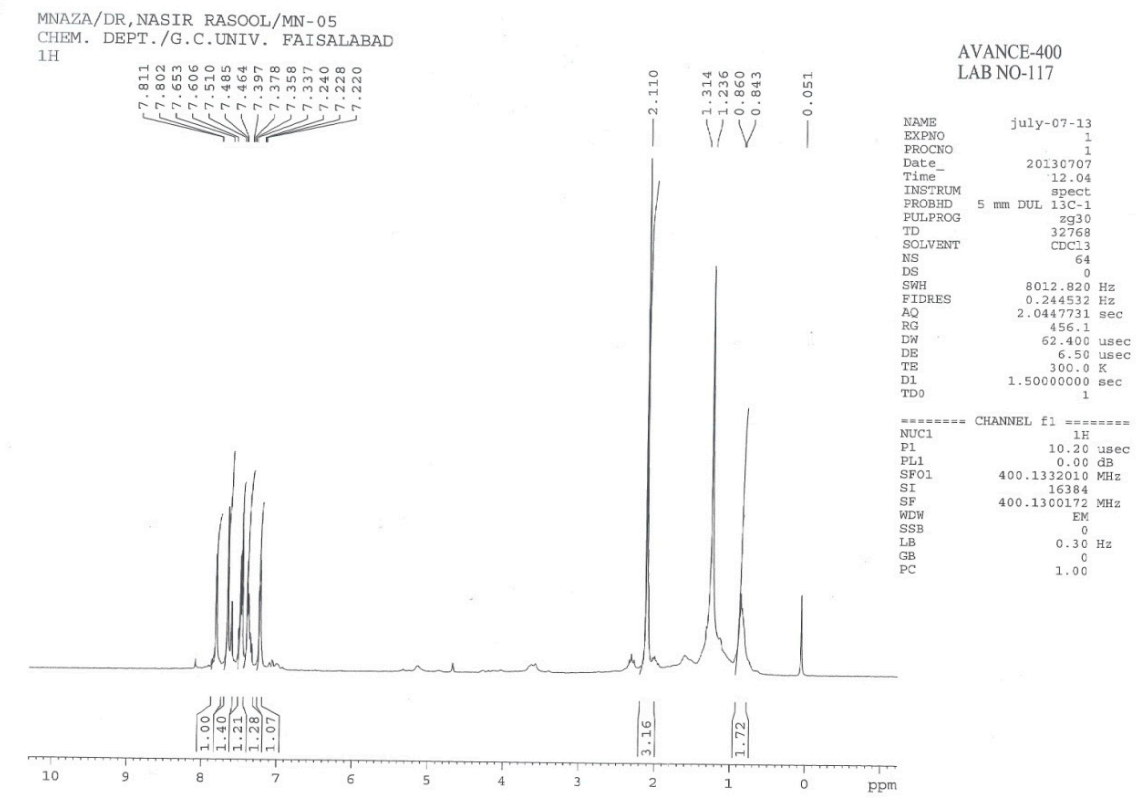

Figure S1. ^{1}H-NMR spectra of compound **4c**.

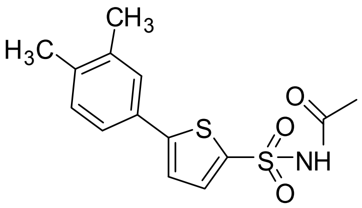

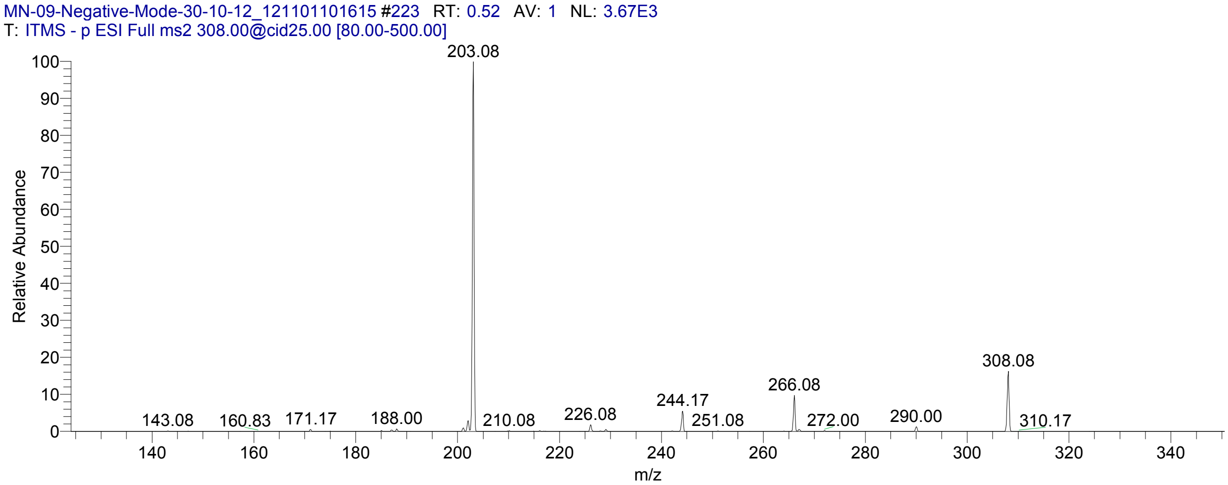

Figure S2. Mass spectrum of compund **4e**.
